# cAMP-induced actin cytoskeleton remodelling inhibits MKL1-dependent expression of the chemotactic and pro-proliferative factor, CCN1

**DOI:** 10.1016/j.yjmcc.2014.11.012

**Published:** 2015-02

**Authors:** Aparna Duggirala, Tomomi E. Kimura, Graciela B. Sala-Newby, Jason L. Johnson, Yih-Jer Wu, Andrew C. Newby, Mark Bond

**Affiliations:** a Bristol Heart Institute, School of Clinical Sciences, University of Bristol, Bristol, BS2 8HW, UK; b Department of Medicine, MacKay Medical College, New Taipei, Taiwan; c Cardiovascular Division, Department of Internal Medicine, MacKay Memorial Hospital, New Taipei, Taiwan

**Keywords:** CCN1, Cyr61, cAMP, 3′-5′-Cyclic adenosine monophosphate, MKL1, VSMC

## Abstract

Elevation of intracellular cAMP concentration has numerous vascular protective effects that are in part mediated via actin cytoskeleton-remodelling and subsequent regulation of gene expression. However, the mechanisms are incompletely understood. Here we investigated whether cAMP-induced actin-cytoskeleton remodelling modulates VSMC behaviour by inhibiting expression of CCN1. In cultured rat VSMC, CCN1-silencing significantly inhibited BrdU incorporation and migration in a wound healing assay. Recombinant CCN1 enhanced chemotaxis in a Boyden chamber. Adding db-cAMP, or elevating cAMP using forskolin, significantly inhibited CCN1 mRNA and protein expression *in vitro*; transcriptional regulation was demonstrated by measuring pre-spliced CCN1 mRNA and CCN1-promoter activity. Forskolin also inhibited CCN1 expression in balloon injured rat carotid arteries *in vivo*. Inhibiting RhoA activity, which regulates actin-polymerisation, by cAMP-elevation or pharmacologically with C3-transferase, or inhibiting its downstream kinase, ROCK, with Y27632, significantly inhibited CCN1 expression. Conversely, expression of constitutively active RhoA reversed the inhibitory effects of forskolin on CCN1 mRNA. Furthermore, CCN1 mRNA levels were significantly decreased by inhibiting actin-polymerisation with latrunculin B or increased by stimulating actin-polymerisation with Jasplakinolide. We next tested the role of the actin-dependent SRF co-factor, MKL1, in CCN1 expression. Forskolin inhibited nuclear translocation of MKL1 and binding of MKL1 to the CCN1 promoter. Constitutively-active MKL1 enhanced basal promoter activity of wild-type but not SRE-mutated CCN1; and prevented forskolin inhibition. Furthermore, pharmacological MKL-inhibition with CCG-1423 significantly inhibited CCN1 promoter activity as well as mRNA and protein expression. Our data demonstrates that cAMP-induced actin-cytoskeleton remodelling regulates expression of CCN1 through MKL1: it highlights a novel cAMP-dependent mechanism controlling VSMC behaviour.

## Highlights

• CCN1 promotes VSMC proliferation, migration and chemotaxis.
• cAMP inhibits CCN1 expression *in vitro* and vascular-injury induced CCN1 expression *in vivo.*
• cAMP induced actin remodelling inhibits CCN1 gene transcription.
• cAMP-induced actin-cytoskeleton remodelling regulates expression of CCN1 via MKL1.
• Highlights a novel cAMP-dependent mechanism controlling VSMC behaviour.

## 1 Introduction

3′-5′ cyclic adenosine monophosphate (cAMP) is a ubiquitous second messenger synthesised in cells by several adenylyl cyclase iso-enzymes in response to vasoactive G_s_ protein-coupled receptor stimulation. cAMP regulates numerous, physiological, cellular functions that are essential for normal blood vessel homeostasis, including proliferation[1], apoptosis[2], differentiation [3], relaxation[4] and inflammation[5]. In vascular smooth muscle cells (VSMC), elevated cAMP levels inhibit mitogen stimulated proliferation *in vitro* [1] and injury induced proliferation *in vivo* [6]. A number of studies have also demonstrated inhibitory effects of cAMP on VSMC migration [7,8], which together with the aforementioned anti-mitogenic properties, reduce intimal lesion formation in experimental vascular injury models [6,9]. Altered or aberrant cAMP signalling has also been implicated in development of many vascular pathologies [10,11], including late vein-graft failure [12–14], angioplasty restenosis [15], atherogenesis [16] and pulmonary hypertension. For example, the ability of VSMC to synthesise cAMP is reduced in response to hypertension [17], vein grafting [14], hypoxia and oxLDL [18], suggesting that deregulation of cAMP signalling contributes towards the development of vascular pathologies.

Activation of the cAMP sensitive protein kinase A (PKA) and subsequent phosphorylation of CREB-dependent gene expression is one well-established mechanism underlying cAMP-dependent cellular responses [19]. However, recent evidence from our laboratory and others implicates actin-cytoskeleton remodelling as an additional key component of cAMP signalling in VSMC [20–23]. Elevated cAMP inhibits the activity of members of the Rho GTPases, which in VSMC [20,23] causes reduced actin polymerisation and acquisition of a ‘stellate’ like morphology, characterised by a loss of actin stress fibres and a condensed cytoplasm. RhoA or Rac1 inhibition using pharmacological inhibitors, siRNA or dominant-negative approaches mimics the effects of cAMP on VSMC morphology, proliferation, migration and relaxation *in vitro* and *in vivo* [20,23], demonstrating the functional role of Rho GTPase inhibition for cAMP responses. Moreover, expression of constitutively-active RhoA or Rac1 mutants effectively prevents cAMP-induced actin cytoskeleton remodelling and reverses many of the biological effects of cAMP in VSMC [20,22,23]. Although inhibition of Rho GTPases and actin polymerisation is an important mechanism underlying cAMP-dependent biological responses in VSMC, the downstream pathways are unclear.

Actin-cytoskeleton remodelling controls expression of multiple genes but it is not clear which, if any, contribute to the effects of cAMP in VSMC. For example, formation of F-actin stress fibres is required in many cell types for efficient mitogen-activated nuclear translocation of ERK1/2 kinases and subsequent early response gene expression [24,25]. In addition, increased levels of actin monomer (G-actin), resulting from impaired actin polymerisation, has also been implicated in modulation of SRF-dependent gene expression, via cytoplasmic sequestration of the SRF co-factors such as MKL1 [26]. Regulation of transcription factor activity via these actin-dependent mechanisms typically controls cell behaviour by rapidly modulating expression of immediate early genes.

To identify genes that are regulated in response to cAMP-mediated repression of RhoA signalling, we cross-referenced our previous cAMP-dependent transcriptomic data [22] with the National Cancer Institute’s pathway interaction database. This identified the Cysteine-rich protein 61 (CCN1) gene as a putative cAMP and RhoA pathway-dependent gene. CCN1 is a ~ 40 kDa secreted protein and member of the CCN family of immediate-response genes that is rapidly induced by mitogens, stress and tissue injury [27]. Importantly, CCN1 expression in various tissues is regulated through mechano-transduction pathways that appear to converge at the level of cytoskeletal actin dynamics [28]. On synthesis, CCN1 is secreted and associates with the ECM or cell surface to modulate a range cell functions, including adhesion, proliferation, migration, inflammation, ECM-remodelling and gene expression [29]. CCN1 is expressed in the vasculature during development where it is essential for vascular integrity [30] and is up regulated in adults in response to vessel injury and in advanced atherosclerotic plaques where it contributes towards monocyte adhesion and intimal lesion formation [31,32]. Here we first inquired whether CCN1 regulated VSMC proliferation and migration. We then investigated the hypothesis that at cAMP elevation might repress expression of CCN1 in VSMC by inhibiting RhoA-mediated actin polymerisation and reduced MKL1-dependent transcription.

## 2 Material and methods

### 2.1 Materials

Male Sprague–Dawley (SD) rats were obtained from Charles River. Culture media and additives were obtained from Invitrogen. All chemicals were obtained from Sigma unless otherwise stated. BAY606583 was from Tocris. Antibodies used were anti-CCN1 (R&D Systems, #AF4055), anti-GAPDH (Millipore, #MAB374), anti-MKL1 (Santa Cruz #sc21558), anti-BrdU (Sigma; #B253).

### 2.2 Smooth muscle cell culture

Male Sprague–Dawley rats were killed by cervical dislocation in accordance with the Directive 2010/63/EU of the European Parliament. Approval was granted by the University of Bristol ethical review board. Medial tissue was carefully dissected from the thoracic aorta and cut into 1 mm^2^ pieces for explant culture, essentially as described previously [33]. Stimulations were performed in 5% foetal calf serum/DMEM unless otherwise stated. Proliferation was measured by culture in the presence of 10 μM bromo-deoxyuridine. Following fixation in 70% ethanol, incorporated bromo-deoxyuridine was detected by immuno-histochemical staining as previously described [21].

### 2.3 Balloon injury of rat carotid arteries and local administration of forskolin

Male Sprague–Dawley rats were obtained from Charles River (UK). The housing and care of the animals and all the procedures used in the study adhered to the guidelines and regulations of Animal Scientific Procedures Act 1986. Balloon injury of left common carotid artery was performed as previously described [34]. Briefly, rats were anesthetised with ketamine (100 mg/kg) and xylazine (5 mg/kg; i.p.) following inhalation of halothane. Left common carotid arteries were injured by three rotating passes with a 2F Forgaty catheter (Actamed, West Yorkshire, UK) introduced through the left external carotid artery. Proximal external carotid arteries were ligated after withdrawal of the balloon catheter. Where indicated, 200 μl of 30% (wt/vol) pluronic gel (F127, Sigma) containing either forskolin (200 μmol/L) or 0.1% DMSO (control) was applied around the injured common carotid arteries (~ 1 cm in length) before closure of the wound. Rats were killed after 7 days using pentobarbital (100 mg/kg i.p.) and vessels collected after perfusion with heparinised PBS or perfusion-fixation with 10% formalin/PBS solution.

### 2.4 Immunohistochemistry

Formalin-fixed, paraffin-embedded arterial sections were deparaffinised, rehydrated and subjected to antigen retrieval (sodium citrate buffer, 0.1 mol/L, pH = 6.0, at 100 °C for 10 min). Sections were blocked with Image-iT FX (Life Technologies) and incubated with 10 μg/ml sheep anti-CCN1 (R&D Systems, #AF4055), biotin-conjugated secondary antibody (Dako), followed by detection with Strepavidin Alexa-Fluor 488.

### 2.5 Migration and chemotaxis assays

VSMC migration was measured using Ibidi culture-inserts with a 500 μm gap width. Inserts were attached to the bottom of 24 well plate wells. Cells were transfected with the indicated siRNAs (as described in Section 2.8) and seeded into both chambers of the Ibidi insert. After 24 h, inserts were removed and cells were allowed to migrate into the exposed wound area for a further 18 h. Cell numbers migrating into the wound area were counted. Chemotaxis assays were performed using a 48 well Neuro Probe chemotaxis chamber fitted with a polycarbonate membrane (8 μm pore size). The lower chamber was filled with serum free media containing the indicated amounts of recombinant CCN1. Serum deprived cells were trypsinised, seeded into the top chamber in serum free medium and allowed to migrate for 6 h. Cells on the top of the membrane were removed with gentle abrasion and the lower side stained with hemotoxylin. Migrated cells were counted and expressed as migrated cells per high power field.

### 2.6 Quantitative RT-PCR and Western blotting

Quantification of mRNA and protein levels was performed by qRT-PCR and Western blotting essentially as described previously [21]. Total RNA, extracted using Qiagen RNeasy kit (Qiagen) was reverse transcribed using QuantiTect RT kit (Qiagen) and random primers. Quantitative PCR was performed using Roche SYBR Green using an Illumina Eco PCR machine (20′@95 °C; 20′@62 °C; 20′@72 °C). Data were normalised to total RNA levels in each reaction. Primers were: rat CCN1 forward 5′-GGA ACT GGC ATC TCC ACA CGA GTT-3′ and reverse 5′-TTG TCC ACA AGG ACG CAC TTC ACA-3′; rat CCN1 pre-spliced hnRNA forward 5′-CAG CTC ACT GAA GAG GCT TCC TGG-3′ and reverse 5′-TAG ACC GGT GCA GAC ACA CAA TGG-3′; rat 36B4 mRNA forward 5′-AGC CAA GGT CGA AGC AAA GGA AGA-3′ and reverse 5′-GAC TTG GTG TGA GGG GCT TAG TCG-3′.

### 2.7 Plasmids, siRNA and adenoviral vectors

A 2.177 kb fragment of the human CCN1 promoter (Hg19;chr1:86044316-chr1:86046493) was amplified from human genomic DNA and cloned into the Kpn1 and Nhe1 restriction sites of pGL4[luc2CP] (Promega). pSRE-Luc luciferase reporter containing five copies of an SRE element was obtained from Agilent. p3XCMVFLAG-wt-MKL and p3XCMVFLAG-MKL1_N100_ vectors expressing wild-type and constitutively-active MKL1 that lacks the N-terminal 100 amino acid actin-binding domain were a gift from Prof. Ron Prywes and have been described previously [35]. An EcoR1 and BamH1 fragment cut from p3XCMVFLAG-MKL1_N100_ was sub-cloned into pDC515 (Microbix) and used to generate the recombinant adenovirus expressing MKL1_N100_. Recombinant adenovirus expressing constitutively active RhoA_(G14V)_ has been described previously [22]. Silencer Select siRNAs targeting rat CCN1 (s135923 and s135925), rat MKL1 (s163756) and rat MKL2 (ABX00O2) were purchased from Life Technolgies. Recombinant CCN1 protein was obtained from Peprotech. Pharmacological MKL inhibitor CCG-1423 was obtained from Cayman Chemical. pCS2 +/hSRF-VP16 plasmid was provided by Alfred Nordheim [36].

### 2.8 Transfection and recombinant adenoviruses transduction

Plasmid transfection was performed using nucleofection. 1 × 10^6^ VSMC were transfected with 3–5 μg of DNA using program A033. Adenoviruses were prepared as previously described [20]. VSMC were infected with adenovirus at 1 × 10^8^ plaque forming units/ml for 16 h.

### 2.9 Reporter gene luciferase assays

Cells were transfected by electroporation with the indicated SRE and CCN1 promoter reporter plasmids together with pTk-Renilla for normalisation. Cells were stimulated with the indicated agents 24 h post-transfection followed by lysis in Promega cell culture lysis buffer. Luciferase and Renilla activity were quantified using the dual reporter assay kit (Promega) according to the manufacturer's instructions using Glomax luminometer (Promega).

### 2.10 Chromatin immunoprecipitation (ChIP) assays

ChIP assays were performed essentially as described previously [37]. ChIP primers used to amplify an amplicon located near the rat CCN1 promoter SRE element were 5′-AAC GCA GGC TAC TGC TAT ACC CAA A-3′ and reverse 5′-CCT GGC TTC TGT TGT GGC GTC TTT T-3′.

### 2.11 Statistical analysis

Statistical analysis was performed using ANOVA with Student–Newman–Keuls post-test or where appropriate a paired Student's t-test. All data are from at least three independent experiments and are presented as mean ± standard error. * indicates p < 0.05, ** indicates p < 0.01, *** indicates p < 0.001.

## 3 Results

### 3.1 CCN1 regulates VSMC proliferation, migration and chemotaxis

**Fig. 1:**
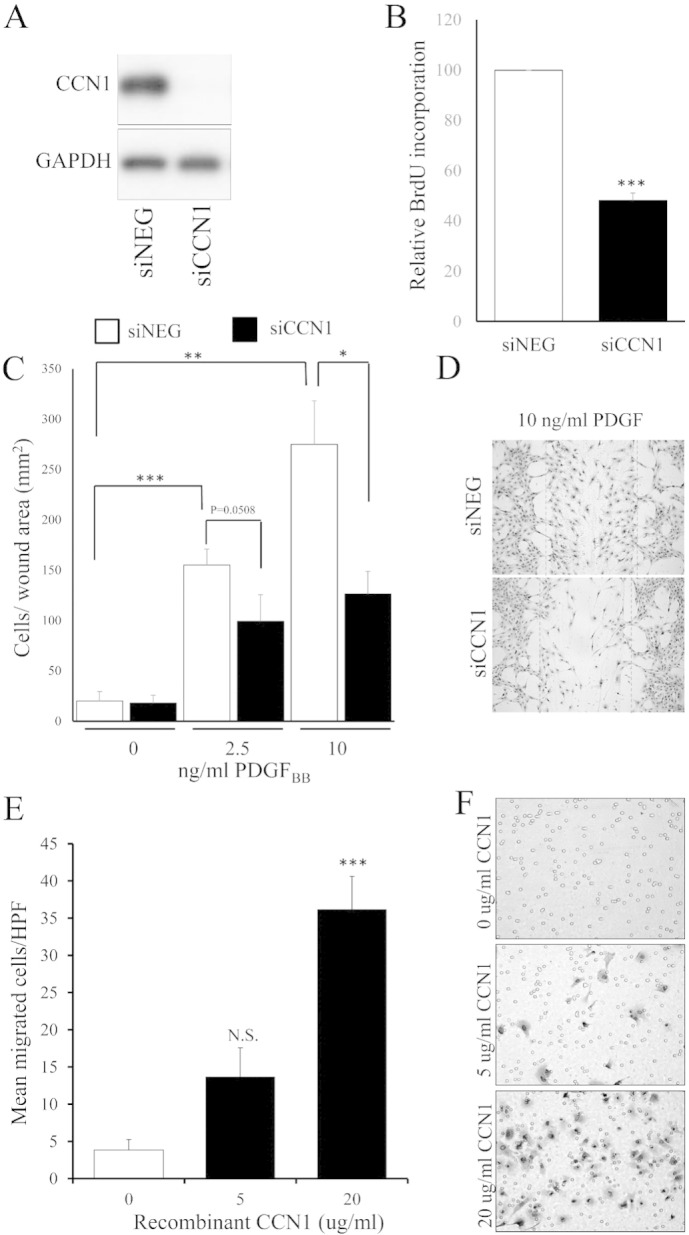
**CCN1 promotes VSMC proliferation, migration and chemotaxis**. Western blot analysis of CCN1 and GAPDH protein 24 h post-transfection with siNEG (□) or siCCN1(∎) (A). Relative BrDU incorporation 24–40 h post-transfection with siNEG of siCCN1; n = 3 (B). Barrier migration assay of siNEG (□) and siCCN1(∎) transfected cells after 18 h stimulation with 2.5 ng/ml or 10 ng/ml PDGF_BB_; n = 6 (C and D). Boyden-chamber chemotaxis assay using 5 or 20 μg/ml recombinant CCN1 in the bottom chamber for 8 h; n = 6 (E and F). □ indicates controls; ∎ indicates stimulated cells. * indicates p < 0.05; ** indicates p < 0.01; *** indicates p < 0.001.

Elevation of intracellular cAMP inhibits proliferation and migration of several cell types, including VSMC [38–40]. Based on this, we hypothesised that cAMP-mediated repression of CCN1 might contribute towards reduced VSMC proliferation and migration. We therefore tested the effects of siRNA-mediated silencing of CCN1 and recombinant CCN1 protein on VSMC proliferation and migration. Transfection with siRNA targeting CCN1 (siCCN1) but not a control siRNA (siNEG) reduced CCN1 protein levels to below the limit of detection without effecting GAPDH protein levels (Fig. 1A). SiCCN1 significantly inhibited proliferation measured by BrdU incorporation to 48.2 ± 2.9% of controls (Fig. 1B). PDGF_BB_ stimulation dose-dependently increased VSMC migration of siNEG transfected cells in *in vitro* wound healing assays (Fig. 1C). CCN1 silencing significantly reduced migration into the wound area induced by 10 ng/ml PDGF_BB_ from 274.9 ± 43.1 cells/wound area to 126.5 ± 22.23 cell/wound area (p < 0.0216; Fig. 1C and D)_._ Furthermore, Boyden chamber chemotaxis assays demonstrated that recombinant CCN1 protein dose dependently stimulated VSMC chemotaxis (Fig. 1E and F).

### 3.2 Elevated cAMP inhibits mitogen-induced expression of CCN1 *in vitro* and injury-induced expression of CCN1 *in vivo*

**Fig. 2:**
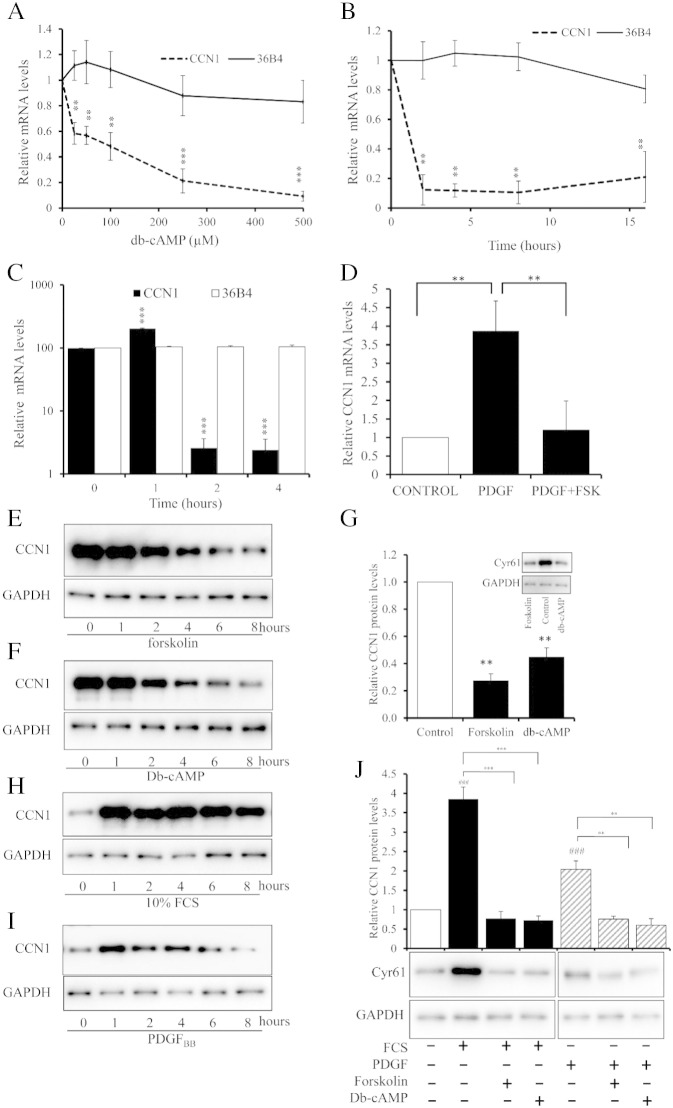
**cAMP inhibits expression of CCN1 in VSMC**. VSMCs were stimulated with for 2 h with the indicated concentrations of db-cAMP; n = 3 (A), with 500 μM db-cAMP for the indicated times; n = 3 (B) or with 25 μM forskolin for the indicated times; n = 3 (C). Quiescent VSMCs were stimulated with 50 ng/ml PDGF_BB_ for 1 h with 25 μM forskolin as indicated; n = 6 (D). Total RNA was analysed for CCN1 (dashed line) and 36B4 mRNA (solid line) using RT-PCR (A–D). Cells were stimulated with 25 μM forskolin (E) or 500 μM db-cAMP (F) for indicated times and CCN1 protein quantified by Western blotting and densitometry at 8 h; n = 3; □ indicates control; ∎ indicates stimulated (G). Quiescent VSMCs were stimulated with 10% FCS (H) or 50 ng/ml PDGF_BB_ (I) for indicated times and CCN1 protein quantified by Western blotting. (J) Quiescent VSMCs were stimulated with 10% FCS (H) or 50 ng/ml PDGF_BB_ for 1 h in the presence of 25 μM forskolin or 500 μM db-cAMP, as indicated. CCN1 protein was quantified by Western blotting. * indicates p < 0.05, ** indicates p < 0.01, *** indicates p < 0.001.

To begin understanding the regulation of CCN1 by cAMP in VSMC, we used the stable cAMP analogue dibutyryl-cAMP (db-cAMP) and the adenylate cyclase activator forskolin. Stimulation with db-cAMP for 2 h significantly and concentration-dependently inhibited CCN1 mRNA levels without effecting mRNA levels of the housekeeping gene, 36B4 (Fig. 2A). Stimulation with 500 μM db-cAMP demonstrated a rapid (by 2 h) and sustained (up to 16 h), significant inhibition of CCN1 mRNA levels (Fig. 2B), while 36B4 mRNA levels remained unaffected. We next used forskolin, to test the role of endogenously produced cAMP. Forskolin stimulation resulted in a transient but significant stimulation of CCN1 mRNA levels at 1 h but a dramatic and highly significant inhibition after 2 h (to 2.6 ± 1.05% of controls) and 4 h (to 2.4 ± 6.6% of controls) (Fig. 2C). Furthermore, forskolin completely reversed the increase in CCN1 mRNA induced by PDGF_BB_ (Fig. 2D). Importantly, db-cAMP and forskolin also reduced CCN1 protein levels as early as 2 h after stimulation (Fig. 2E and F). The inhibition by db-cAMP (to 44.7 ± 6.9% of control; p < 0.01) and forskolin (to 27.3 ± 5.2% of controls; p < 0.01) was significant after 8 h (Fig. 2G). Stimulation of quiescent cells with 10% serum mitogens resulted in a rapid (3.5 ± 0.3 fold; p < 0.001 after 1 h) and sustained (up to 8 h) increase in CCN1 protein levels (Fig. 2H and J). Stimulation with PDGF_BB_ resulted in a weaker but still significant (2 ± 0.22 fold at 1 h; p < 0.01) and transient up-regulation of CCN1 protein (Fig. 2I and J). Importantly, db-cAMP and forskolin both completely reverse the increase in CCN1 protein levels induced by either 10% serum or PDGF_BB_ (Fig. 2J). A similar forskolin-mediated inhibition of CCN1 mRNA and protein was detected in human aortic VSMC (Figure S1). Taken together, these data demonstrate that cAMP-signalling rapidly inhibits the basal and mitogen induced levels of CCN1 mRNA and protein expression in rat and human VSMC.

**Fig. 3:**
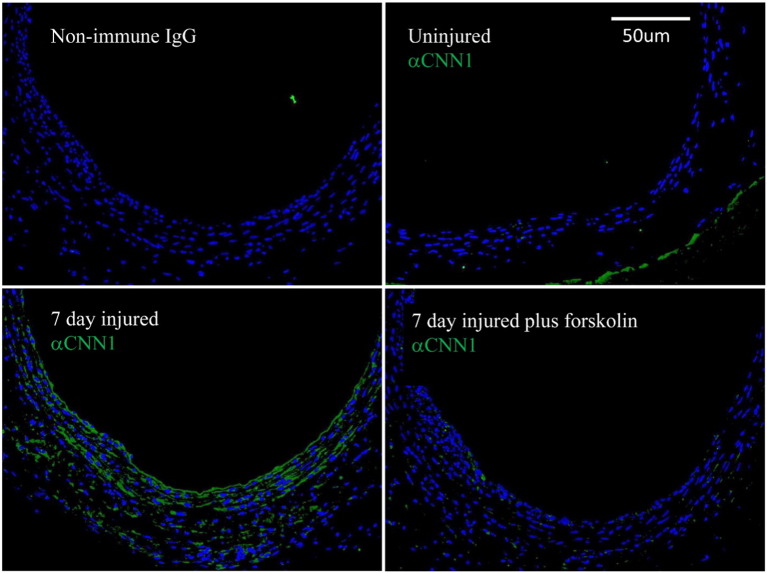
**Forskolin inhibits balloon-injury induced expression of CCN1 in rat carotid arteries *in vivo*.** Immunohistochemical staining for CCN1 in balloon injured rat carotid arteries treated with forskolin. Immediately after balloon injury arteries were surrounded by 200 μl of 30% pluronic gel with or without forskolin. CCN1 (green) expression was analysed by immunohistochemistry in sections of either uninjured rat carotids or carotid arteries 7 days after balloon injury. Nuclei are stained blue with DAPI. Scale bar = 50 μm.

As a first step to investigate the regulation of CCN1 in VSMC *in vivo*, we studied the effect of peri-adventitial application of forskolin on expression of CCN1 protein in rat carotid arteries after balloon injury. CCN1 protein was undetectable in uninjured rat carotid arteries (Fig. 3) but strongly up regulated in medial cells 7 days post-injury. Importantly, forskolin treatment immediately post-injury completely prevented injury-induced expression of CCN1 (Fig. 3).

### 3.3 Adenosine and Cicaprost inhibit CCN1 expression

**Fig. 4:**
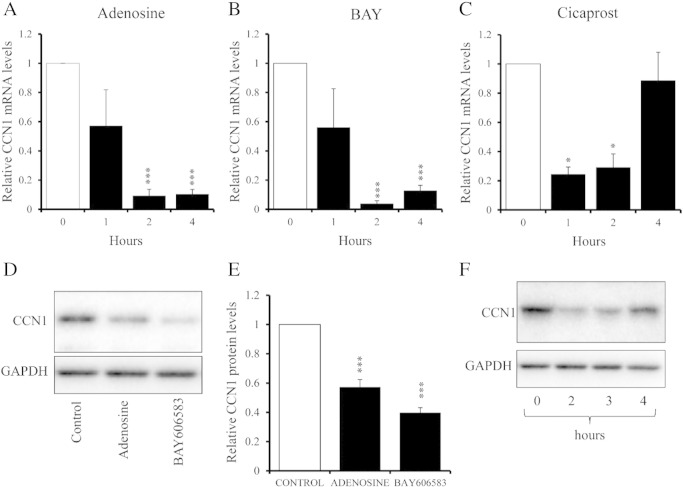
**Inhibition of CCN1 expression by Adenosine, BAY60-6582 and Cicaprost**. Cells were stimulated with 100 μM adenosine; n = 3 (A, D, E), 1 μg/ml BAY-60-6583; n = 3 (B, D, E) or 1 μM Cicaprost; n = 4 (C and F) for indicated times and analysed for CCN1 mRNA (A–C) and protein (D–F) expression qRT-PCR and Western blotting. * indicates p < 0.05, ** indicates p < 0.01, *** indicates p < 0.001. □ indicates control; ∎ indicates stimulated.

Vasoactive agents, including adenosine and prostacyclin stimulate cAMP production via activation of G-protein coupled receptors (GPCRs), thus modulating VSMC function physiologically. To test whether activation of these GPCRs inhibits CCN1 expression we stimulated VSMC either with the GPCR agonists, adenosine, the specific adenosine A2B-receptor agonist BAY60-6583 or the prostacyclin mimetic, Cicaprost. Adenosine and BAY60-6583 significantly inhibited CCN1 mRNA levels after 2 and 4 h (Fig. 4A and B) and inhibited CCN1 protein levels after 4 h (Fig. 4D and E). Cicaprost also transiently inhibited CCN1 mRNA and protein levels at 1 and 2 h, returning to control levels after 4 h (Fig. 4C and F).

### 3.4 Elevation of cAMP inhibits CCN1 transcription

**Fig. 5:**
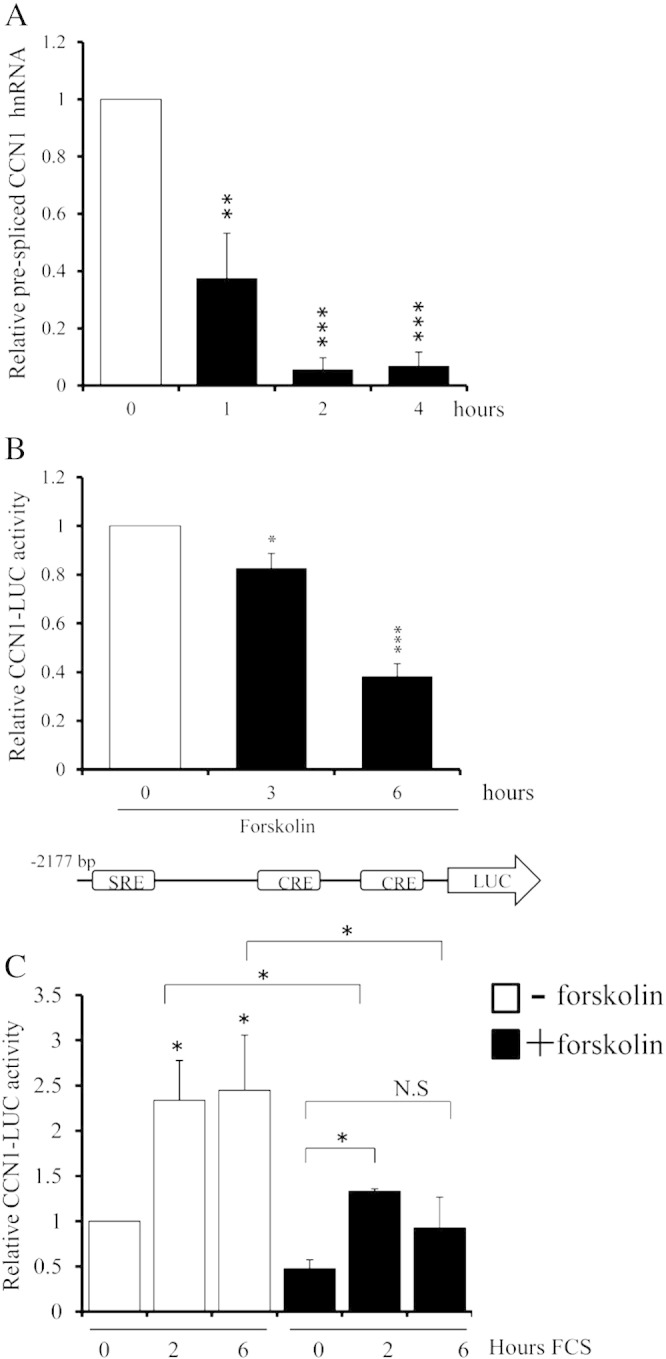
**Forskolin inhibits CCN1 gene transcription.** Cells were stimulated with 25 μM Forskolin for indicated times (A, B). CCN1 pre-spliced hnRNA quantified by qRT-PCR; n = 3 (B). Luciferase activity was quantified in cells transfected with CCN1-LUC; n = 6 (B). Quiescent cells were stimulated with 10% FCS for indicated in the presence or absence of 25 μM forskolin; n = 3 (C). * indicates p < 0.05, ** indicates p < 0.01, *** indicates p < 0.001 vs. Control. □ indicates control; ∎ indicates forskolin stimulated cells.

To test whether elevated cAMP reduces CCN1 mRNA levels by inhibiting CCN1 gene transcription we quantified CCN1 hnRNA (pre-spliced RNA), a surrogate measure of transcriptional rate [41], and activity of a − 2177 bp CCN1 promoter luciferase reporter (CCN1-LUC). Forskolin treatment potently inhibited levels of CCN1 hnRNA within 1 h (to 37.5 ± 15.6% of controls; p < 0.01) with maximal inhibition after 2 h (to 5.7 ± 4.0% of control; p < 0.001; Fig. 5A) and also inhibited basal CCN1-LUC promoter activity after 3 and 6 h (Fig. 5B). Inhibition of CCN1-LUC reporter activity was slower than endogenous hnRNA, presumably due to inherent differences in turnover rates of luciferase protein and CCN1 mRNA. Addition of serum mitogens to quiescent cells significantly stimulated CCN1-LUC activity after 2 h (2.34 ± 0.43 fold; p > 0.05) and 6 h (2.45 ± 0.61 fold; p < 0.05); and this induction was significantly reduced (p < 0.05) in the presence of forskolin (Fig. 5C). Taken together, these data indicate that mitogens increase and cAMP elevation inhibits CCN1 gene transcription.

### 3.5 Elevation of cAMP suppresses CCN1 expression via inhibition of RhoA

**Fig. 6:**
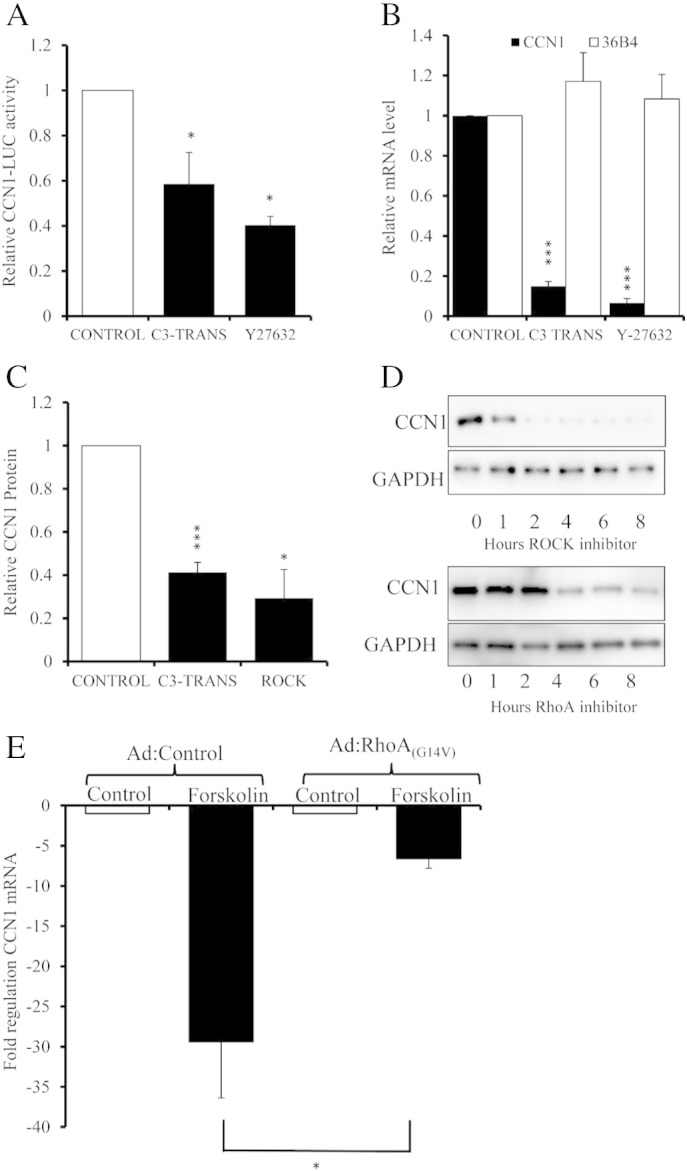
**Forskolin regulates CCN1 expression via the RhoA-ROCK pathway.** Cells were stimulated with 2 μg/ml C3-transferase or 10 μM Y27632 (A–D). Luciferase activity was quantified after 6 h in cells transfected with CCN1-LUC; n = 3 (A). CCN1 mRNA was quantified after 4 h by qRT-PCR; n = 3 (B) and CCN1 protein after 8 h; n = 5 for C3-transferase and n = 3 for ROCK inhibitor (C) or the indicated times (D). Cells were infected with either Ad:Control or Ad:Rho_(G14V)_ and stimulated with 25 μM forskolin for 2 h, 24 h post-infection (E). CCN1 mRNA was quantified using qRT-PCR; n = 6. * indicates p < 0.05, ** indicates p < 0.01, *** indicates p < 0.001 vs. Control. □ indicates control; ∎ indicates stimulated cells.

Elevation of cAMP is known to inhibit that activity of RhoA [22,23], which is involved in cAMP-induced morphological changes and actin-cytoskeleton remodelling. We therefore asked whether RhoA and its effector, ROCK are involved in the regulation of CCN1 expression by cAMP in VSMC. Inhibition of RhoA or ROCK activity for 6 h using C3-transferase or Y27632, respectively, resulted in a significant inhibition (to 58.9 ± 13.9%, p < 0.05 for C3 transferase and to 40.3 ± 3.9%, p < 0.05 for Y27632) of CCN1-LUC activity (Fig. 6A). C3-transferase and Y27632 also significantly inhibited CCN1 mRNA levels (to 15.0 ± 2.3%, p < 0.001 for C3 transferase and to 6.7 ± 2.2%, p < 0.001 for Y27632) after 4 h (Fig. 6B) without effecting 36B4 mRNA levels. CCN1 protein levels were similarly reduced (to 41.3 ± 4.5%, p < 0.001) by C3 transferase and Y27632 (to 29.3 ± 13.4%, p < 0.05) after 8 h (Fig. 6C and D). We next infected cells with a recombinant adenovirus expressing a constitutively active mutant of RhoA (Ad:RhoA_G14V_) to confirm a functional role for RhoA signalling. Forskolin stimulation resulted in a 29.4 ± 7.0 fold inhibition of CCN1 mRNA expression in control virus infected cells (Ad:Control; Fig. 6E). Infection with Ad:RhoA_G14V_ did not significantly alter basal CCN1 mRNA levels. However, forskolin stimulation only resulted in a 6.6 ± 1.18 fold inhibition of CCN1 mRNA in cells infected Ad:RhoA_G14V,_ representing a significant rescue of CCN1 expression by constitutively active RhoA.

### 3.6 cAMP inhibits CCN1 expression via monomeric actin

**Fig. 7:**
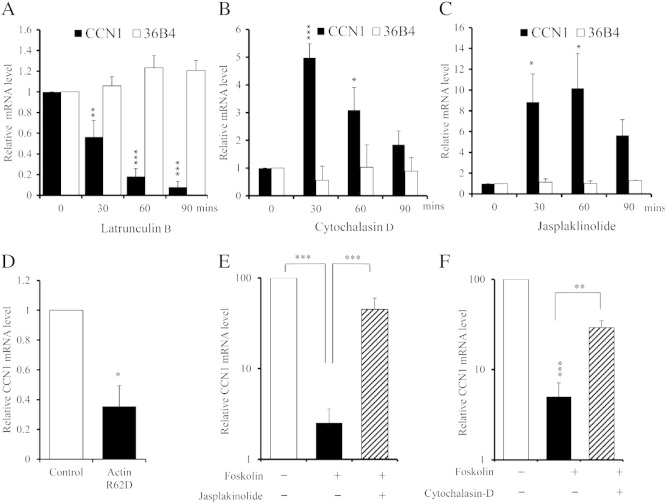
**Forskolin inhibits CCN1 via a monomeric actin-dependent mechanism**. Cells were treated with 5 μg/ml latrunculin-B (A), 2 μM cytochalasin-D (B) or 0.5 μM jasplakinolide (C) for the indicated times; n = 3. CCN1 (∎) and 36B4 (□) mRNA were quantified using qRT-PCT (A–C). Cells were infected with either Ad:Control (□) or Ad:Actin_(R62D)_ (∎) and CCN1 mRNA levels were quantified 24 h post infection; n = 5 (D). Cells were stimulated with 25 μM forskolin for 90 min either in the presence or absence of 0.5 μM jasplakinolide (E) or 2 μM cytochalasin-D (F) and CCN1 mRNA levels quantified; n = 4. * indicates p < 0.05, ** indicates p < 0.01, *** indicates p < 0.001 vs. Control.

Repression of RhoA activity mediated by cAMP inhibits actin polymerisation [23,22], resulting in a loss of F-actin stress fibres (F-actin) and a concomitant increase in actin monomer (G-actin). We used various actin binding drugs that inhibit or promote actin polymerisation to test directly the role of actin polymerisation in regulation of CCN1 expression. Latrunculin-B blocks actin polymerisation, thus reducing F-actin and increasing G-actin. Latrunculin-B treatment rapidly inhibited (to 56.5 ± 15.9% after 30 min; p < 0.01) CCN1 mRNA, with maximal inhibition after 90 min (to 8.0 ± 5.4%; p < 0.001; Fig. 7A). Cytochalasin-D inhibits actin polymerisation, thus reducing F-actin levels and increasing G-actin. In contrast to latrunculin-B, cytochalasin-D also sequesters actin monomer, effectively reducing its bio-availability to actin binding proteins. Interestingly, cytochalasin-D treatment resulted in a transient but significant stimulation (5.0 ± 0.51 fold after 30 min; p < 0.001 and 3.1 ± 0.81 fold after 60 min; p < 0.05) of CCN1 mRNA levels (Fig. 7B). Jasplakinolide, which promotes actin polymerisation, rapidly stimulated (8.8 ± 2.7 fold after 30 min; p < 0.05 and 10.2 ± 3.3 fold after 60 min; p < 0.05) CCN1 mRNA, without effecting 36B4 mRNA levels (Fig. 7C). Taken together, these data imply that CCN1 mRNA is inhibited by elevated levels of monomeric actin (G-actin), instead of being dependent on the presence of polymerised actin. To test this hypothesis further, we elevated monomeric actin levels in VSMC using a recombinant adenovirus expressing a polymerisation defective mutant of actin (Ad:Actin_R62D_). Infection with Ad:Actin_R62D_ resulted in a significant inhibition (to 35.3 ± 14%; p < 0.05) of CCN1 mRNA levels, compared to Ad:Control infected cells, consistent with monomeric actin mediated repression of CCN1 expression (Fig. 7D). We next sought to test whether cAMP-mediated inhibition of CCN1 was dependent on monomeric actin. For these experiments, free monomeric actin was reduced either by polymerisation with jasplakinolide or by sequestration with cytochalasin-D. In agreement with our earlier data, forskolin significantly inhibited CCN1 mRNA levels (to 2.5 ± 1.1% of controls; p < 0.001) in untreated cells. However, co-stimulation with jasplakinolide significantly (p < 0.05) attenuated the inhibitory effects of forskolin (Fig. 7E). Likewise, co-stimulation with cytochalasin-D also significantly attenuated forskolin's inhibitory effects (from 5.03 ± 2.07% of controls for forskolin to 29.17 ± 5.7% of controls for forskolin plus cytochalasin-D; p < 0.01; Fig. 7F). Taken together, these data indicate that cAMP represses CCN1 expression by depolymerising actin fibres and increasing the concentration of monomeric actin. However, the downstream mechanism linking actin monomers to CCN1 expression remained unknown.

### 3.7 cAMP-dependent regulation of CCN1 via MKL and SRF

**Fig. 8:**
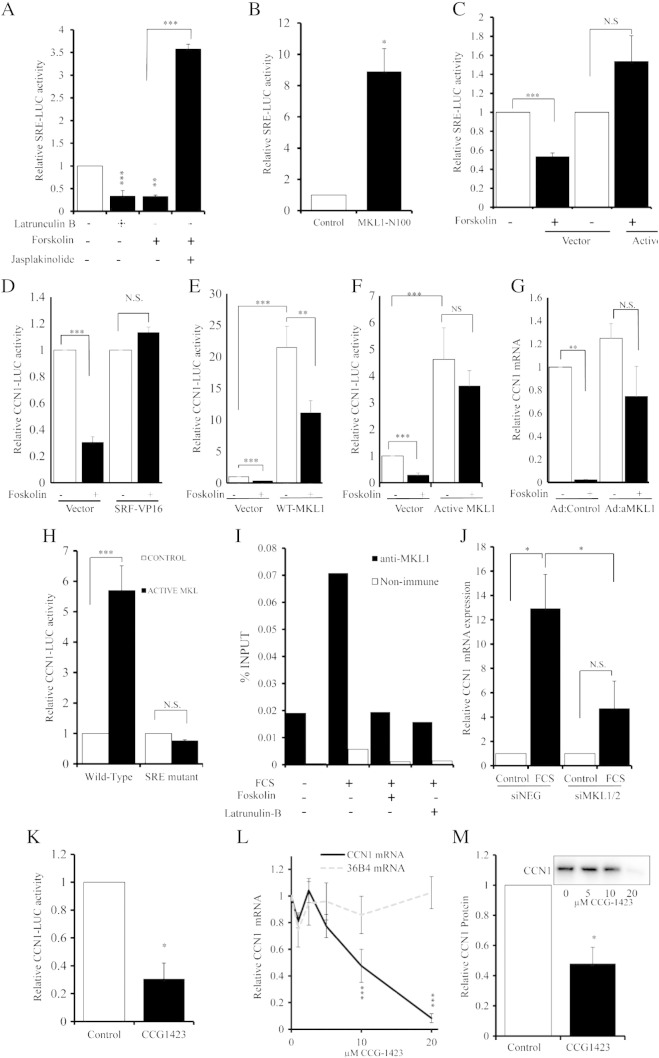
**Forskolin-inhibits CCN1 expression via an MKL1 and SRF-dependent mechanism**. SRE-LUC activity in cells stimulated for 6 h with 5 μg/ml latrunculin B, 25 μM forskolin of 0.5 μM jasplaskinolide, as indicated; n = 3 (A). SRE-LUC activity in cells transfected with active MKL1_N100_ or control vector; n = 4 (B). SRE-LUC activity in control vector or MKL_N100_ transfected cells stimulated with 25 μM forskolin for 6 h; n = 3 (C). CCN1-LUC activity in control vector or SRF-VP16 transfected cells stimulated with 25 μM forskolin for 6 h; n = 3 (D). CCN1-LUC activity in control vector or WT-MKL transfected cells stimulated with 25 μM forskolin for 6 h; n = 3 (E). CCN1-LUC activity in control vector or MKL_N100_ transfected cells stimulated with 25 μM forskolin for 6 h; n = 3 (F). CCN1 mRNA in cells infected with control or MKL_N100_ adenoviruses stimulated with 25 μM forskolin for 2 h; n = 3 (G). Wild-type and SRE-mutated CCN1-LUC activity in cells infected with control or MKL_N100_ adenoviruses; n = 5 (H). ChIP analysis of MKL1 binding to the distal CCN1 promoter after stimulation with 10% FCS, 25 μM forskolin or 5 μg/ml latrunculin B for 20 min (I). CCN1 mRNA levels in cells transfected with siNEG or siMKL1/2 and stimulated with 10% FCS (black bars) for 2 h; n = 4 (J). Effect of 18 h stimulation with 20 μM CCG1423 on CCN1-LUC activity; n = 3 (K). Effect of indicated doses of CCG1423 for 18 h on CCN1 mRNA; 1804n = 5 (L). Effect of indicated doses of CCG1423 for 18 h on CCN1 protein levels; n = 3 (M). White bars indicate unstimulated controls while black bars indicate stimulated conditions, unless otherwise indicated. * indicates p < 0.05, ** indicates p < 0.01, *** indicates p < 0.001 vs. Control. N.S. indicates no significant difference.

Monomeric actin has been shown in fibroblasts [26] to inhibit expression of specific subsets of SRF-dependent genes via cytoplasmic sequestration of the SRF co-factors MKL1 and 2 [26,42]. We asked whether cAMP regulates CCN1 expression in VSMC via this mechanism. We initially tested whether forskolin modulates, in an actin dependent manner, SRE-dependent transcriptional activity measured with an SRE-LUC reporter construct. Forskolin stimulation significantly inhibited (to 32.9 ± 3.0%; p < 0.01) SRE-dependent transcriptional activity (Fig. 8A). Inhibition of actin polymerisation with latrunculin-B also significantly repressed SRE activity (to 34.3 ± 11.4%; p < 0.001). Importantly, forced actin polymerisation with jasplakinolide completely prevented foskolin-mediated inhibition of SRE activity, consistent with actin-dependent regulation of SRE activity by forskolin (Fig. 8A). We next tested the role of MKL1 in forskolin inhibition of SRE-activity using a constitutively active truncation of MKL1 (MKL1_N100_). This mutant lacks the N-terminal 100 amino acids containing the actin-binding RPEL domain and hence promotes SRE-activity in an actin-independent manner. Consistent with this, MKL1_N100_ expression significantly enhanced (by 8.9 ± 1.5 fold; p < 0.05) SRE-LUC activity (Fig. 8B). More importantly, MKL1_N100_ completely reversed forskolin-mediated inhibition of SRE-LUC activity (Fig. 8C), implying that cAMP regulates SRE-activity via an MKL1-dependent mechanism. We therefore asked whether the conserved SRE-element (at − 2060 bp; Figure S2) explains cAMP-dependent regulation of CCN1 expression. Consistent with this possibility, expression of constitutively-active SRF (SRF-VP16) completely prevented forskolin-mediated inhibition of CCN1 promoter activity (Fig. 8D. Expression of wild-type MKL1 (WT-MKL1) significantly stimulated basal CCN1 promoter activity (Fig. 8E) but forskolin-stimulation of WT-MKL1 expressing cells still significantly inhibited CCN1 promoter activity (Fig. 8E). Moreover, expression of active MKL1 (MKL1_N100_) also significantly stimulated CCN1 promoter activity (Fig. 8F). However, MKL1_N100_ expression completely blocked forskolin-mediated inhibit of CCN1 promoter activity (Fig. 8F) and mRNA expression (Fig. 8G). Most importantly, MKL1_N100_-dependent activation of the CCN1 promoter (5.7 ± 0.81 fold; p < 0.01) was abolished by mutation of the distal SRE-element (Fig. 8H). Furthermore, forced actin polymerisation with jasplakinolide significantly stimulated activity (2.9 ± 0.65 fold; p < 0.01) of the wild-type but not SRE-mutated CCN1 promoter (Figure S3), linking both actin-dependent and MKL1-dependent regulation of CCN1 expression to this SRE promoter element. We next asked whether forskolin-mediated inhibition of CCN1 expression is associated with reduced binding of endogenous MKL1 to the CCN1 promoter using chromatin immuno-precipitation assays (ChIP). ChIP analysis demonstrated that serum stimulated binding of endogenous MKL1 to this promoter region; and that binding was inhibited by co-stimulation with forskolin or latrunculin-B (Fig. 8I). The functional significance of endogenous MKL1/2 for CCN1 expression was confirmed using siRNA-mediated silencing and the pharmacological MKL inhibitor, CCG-1423. Transfection with MKL 1 and 2 siRNA significantly suppressed endogenous MKL1 and MKL2 mRNA levels 24 h post transfection (Figure S4). Serum stimulation significantly elevated CCN1 mRNA levels in siNEG but not siMKL1/2 transfected cells (Fig. 8J). Furthermore, treatment with CCG-1423 significantly inhibited CCN1 promoter activity (Fig. 8K), mRNA (Fig. 8L) and protein expression (Fig. 8M). Taken together, these data demonstrate that mitogens increase and cAMP represses SRE-dependent expression of CCN1 by inhibiting MKL1 binding to the distal SRE-element.

## 4 Discussion

Increased migration and proliferation of VSMC are major factors that contribute towards the pathological intimal thickening underlying the development of numerous vascular pathologies, including late vein graft failure, in-stent restenosis, pulmonary artery hypertension and atherosclerosis. Current models of disease development posit a complex interaction of phenotypically modulated VSMC with growth factors and other extracellular mediators produced either in an autocrine manner or from nearby endothelial or inflammatory cells. Consistent with this, both migration and proliferation of VSMCs are triggered by combined actions of growth factor and other (e.g. Wnt) signalling pathways, and also depend on permissive cell–matrix and cell–cell interactions [43]. Consequently, the number of essential mediators that have been identified continues to rise, despite decades of research. Our present findings (Fig. 1) support an additional permissive role for the matri-cellular protein CCN1 in VSMC migration and proliferation. CCN1 is a ~ 40 kDa a secreted matricellular factor and member of the CCN family of immediate-response genes. On synthesis, CCN1 is secreted and associates with the ECM or cell surface via specific integrin binding and heparin sulphate proteoglycans to modulate intracellular signalling [27]. In addition, CCN1 may also directly inhibit BMP signalling or displace ECM-bound bFGF, thus enhancing bFGF-induced proliferation [29,44]. CCN1 has been previously implicated in a range cell functions, including cell adhesion [45], proliferation, migration [45], inflammation, ECM-remodelling and gene expression [46]. CCN1 has also been implicated in the development of various cardiovascular pathologies. In the heart, CCN1 expression is elevated in patients with myocarditis, dilated cardiomyopathy and myocardial infarction [47,48]. CCN1 is also up-regulated in rodent models of myocardial infarction, atherosclerosis [32], diabetic retinopathy [49], hypertension [50] and most importantly for this study, angioplasty restenosis [31]. In this context, lentiviral mediated knockdown of CCN1 was shown to suppress VSMC proliferation and neoinitmal lesion development *in vivo* 14 days after balloon injury to the rat carotid artery [31].

The focus of our present study was not to define further the production and action of CCN1 but to investigate its down-regulation by cAMP because interruption of the vicious cycle of VSMC migration, proliferation and disease progression has the potential limit pathological intima formation. Consistent with this, a large body of evidence documents the ability of cAMP to inhibit VSMC migration and proliferation and ultimately repress intima formation physiologically *in vivo* [6]. However, the early cAMP-dependent signalling mechanisms underlying these vascular protective effects are incompletely characterised and this has probably hampered the development of clinically useful inhibitors. Elevation of cAMP acts on multiple targets to inhibit the early growth factor and matrix-related signalling pathways. For example, decreasing levels of Skp2 and increasing those of the cyclin-dependent kinase inhibitors p27^Kip1^ and p21^cip1^ are of significance for regulating the cell cycle in VSMC [6,33]; but these are delayed effects and by no means direct targets of cAMP or the only important mechanisms. Our new data demonstrate that decreased production of CCN1 occurs rapidly after cAMP elevation and is an important factor regulating VSMC proliferation and migration.

**Fig. 9:**
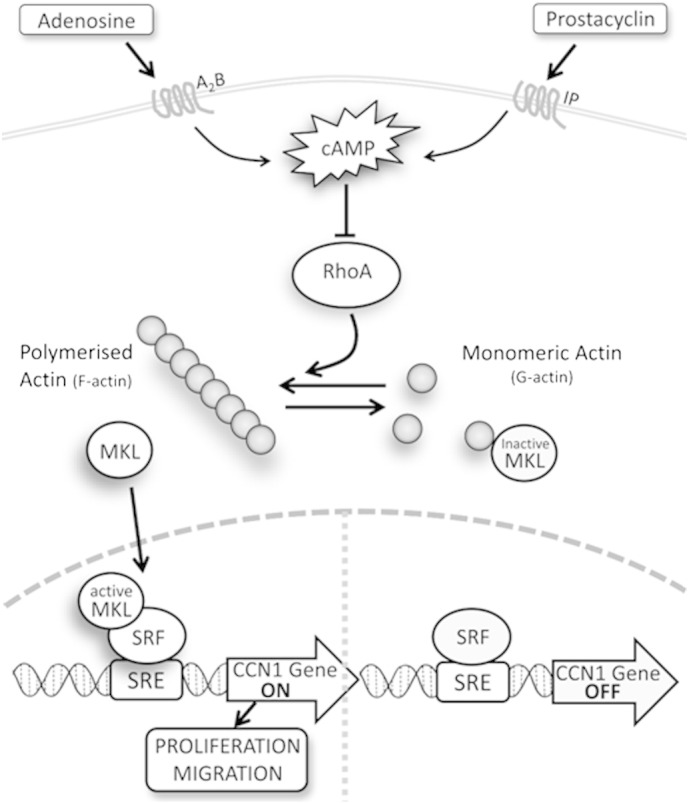
**Proposed mechanism underlying cAMP-dependent regulation of CCN1 in VSMC.** cAMP signalling rapidly inhibits the expression of CCN1 in VSMC. Elevation of cAMP inhibits CCN1 expression via inhibition of RhoA and hence disruption of the actin cytoskeleton. The resulting increase in monomeric actin leads to inhibition of MKL1-dependent SRF transcriptional activity and reduces CCN1. Reduced expression of CCN1 contributes towards inhibition of proliferation and migration.

Defining the precise mechanisms underlying the actions of cAMP in VSMC could have an important impact on development of pharmacological approaches. Recent evidence from our laboratory and others has emphasised the central role played by actin cytoskeleton remodelling for many of the effects of cAMP. Elevated cAMP inhibits the activity of members of the Rho GTPases, including RhoA and Rac1 [20,22,23], rapidly inhibiting actin polymerisation and inducing in cultured VSMC a ‘stellate’ morphology, characterised by a condensed cytoplasm and loss of actin stress-fibres. Precisely how these cAMP induced actin changes modulate VSMC behaviour has remained poorly understood. In this study, we describe a novel pathway that culminates in suppression of CCN1 transcription (Fig. 9). Our new findings elucidate each step of this pathway by showing that cAMP elevation: (i) rapidly inhibits mitogen induced transcription of the CCN1 gene *in vitro* and vascular injury induced expression of CCN1 *in vivo*, (ii) represses CCN1 expression by inhibiting the RhoA/ROCK pathway and disrupting actin polymerisation, (iii) inhibits CCN1 transcription dependent on generation of monomeric actin rather than loss of actin fibres, and (iv) inhibits MKL1-dependent regulation of the distal SRE-element in the CCN1 promoter.

Taking each of these steps in turn, we first demonstrated that elevated cAMP potently and rapidly inhibits expression of CCN1 at the level of pre-spliced mRNA, steady-state mRNA and protein (Fig. 2). Furthermore, this occurred in response to stable cAMP analogues, adenylate cyclase activation with forskolin or simulation with physiological GPCR agonists that act via cAMP, including adenosine and prostacyclin. We showed that arterial balloon injury *in vivo* induces CCN1 expression and that this is inhibited by forskolin. We previously demonstrated that cAMP inhibits activity of several Rho GTPases [21,22]. We show here that this mechanism is responsible for the inhibition of CCN1 gene transcription because it can be mimicked by pharmacological inhibition of RhoA or ROCK and reversed by expression of a constitutively-active RhoA mutant. These observations are consistent with previously published studies showing, for example, that thrombin or mechanical strain induced expression of CCN1 is dependent on RhoA activation [51,52]. Rho GTPase activity is linked to cytoskeletal remodelling via well characterised mechanisms and our new data demonstrate that RhoA-mediated cytoskeletal remodelling underlies cAMP-dependent regulation of CCN1 expression. Blocking actin polymerisation with latrunculin-B potently inhibited CCN1 expression, whereas stimulating actin polymerisation with jasplakinolide increased CCN1 expression. It remained to be demonstrated whether CCN1 expression depended on the presence of actin polymer (F-actin) or was inhibited by elevated levels of actin monomer (G-actin). Our data strongly suggest that latter mechanism. For example, cytochalasin-D, which inhibits actin polymerisation but also sequesters actin–monomer, stimulated rather than inhibited CCN1 expression under baseline conditions. Consistently, forced expression of a non-polymerisable actin mutant also inhibited CCN1 expression under baseline conditions. Moreover, reducing the availability of free actin monomer with either cytochalasin-D or Jasplakinolide reversed the inhibitory effects of forksolin on CCN1 expression. Taken together, these results demonstrate that the cAMP-dependent increase in monomeric actin drives inhibition of CCN1 transcription.

Increased levels of monomeric-actin, resulting from impaired actin polymerisation, have been implicated in sequestration of the SRF co-factor MKL1 and therefore blocking SRF-dependent regulation of immediate-early gene expression [53]. Consistent with this, we previously demonstrated that cAMP inhibits SRF-dependent transcriptional activity in VSMC and that forskolin inhibits nuclear translocation of MKL1 into the nucleus [22]. For this study, we noted that the CCN1 promoter contains a conserved SRE-binding element and went on to provide convincing data implicating MKL1 in cAMP-mediated inhibition of CCN1. In detail our current data demonstrated that MKL-silencing inhibited CCN1 expression and cAMP reduced binding of MKL1 to the CCN1 promoter. Furthermore, the constitutively active mutant of MKL1 (MKL1_N100_), lacking the N-terminal actin-binding RPEL domain, completely prevented forskolin-mediated reduction in CCN1 expression. Furthermore, we showed by mutation analysis that the ability of MKL1 and actin polymerisation to regulate CCN1 expression is absolutely dependent on the distal SRE promoter element. The implication of these data is that cAMP-mediated inhibition of RhoA and the subsequent reduction in actin polymerisation leads to reduced MKL1-SRF-dependent CCN1 expression (Fig. 9). Consistent with this, forced actin polymerisation with Jasplakinolide or actin monomer sequestration with cytochalasin-D blocks the effects of forskolin on SRE transcriptional activity or CCN1 promoter activity.

### 4.1 Conclusions

In summary, this study demonstrates that CCN1 stimulates VSMC proliferation, migration and chemotaxis. Moreover, it shows for the first time that cAMP signalling rapidly inhibits the expression of CCN1 in VSMC *in vitro* and *in vivo*. Elevation of cAMP inhibits CCN1 expression via inhibition of RhoA and hence disruption of the actin cytoskeleton. The resulting increase in monomeric actin leads to inhibition of MKL1-dependent SRF transcriptional activity and reduces CCN1 transcription. This represents a novel cAMP-sensitive signalling pathway (Fig. 9) that controls the expression of CCN1 and very likely other SRE-dependent immediate response genes also. Our study highlights the previously underappreciated importance of actin-cytoskeleton remodelling in mediating the biological effects of cAMP in VSMC and suggests several new targets for pharmacological intervention

## Disclosures

None

## Glossary

BrdU: 5-Bromo-2-deoxyuridine
cAMP: 3′-5′-Cyclic adenosine monophosphate
CREB: cAMP response element binding protein
SRE: Serum response element
SRF: Serum response factor
VSMC: Vascular smooth muscle cell
RhoA: Ras homolog family member A
Rac: Ras-related C3 botulinum toxin substrate 1
VSMC: Vascular smooth muscle cell
