# Supplementary material for: cAMP-induced actin cytoskeleton remodelling inhibits MKL1-dependent expression of the chemotactic and pro-proliferative factor, CCN1
Source: J Mol Cell Cardiol. 2015 Feb;79:157–68. doi: 10.1016/j.yjmcc.2014.11.012 (PMC4312355; doi:10.1016/j.yjmcc.2014.11.012)
Supplement: Supplementary file 1 — Supplementary figures [file mmc1.pdf]

Supplement Figure S1

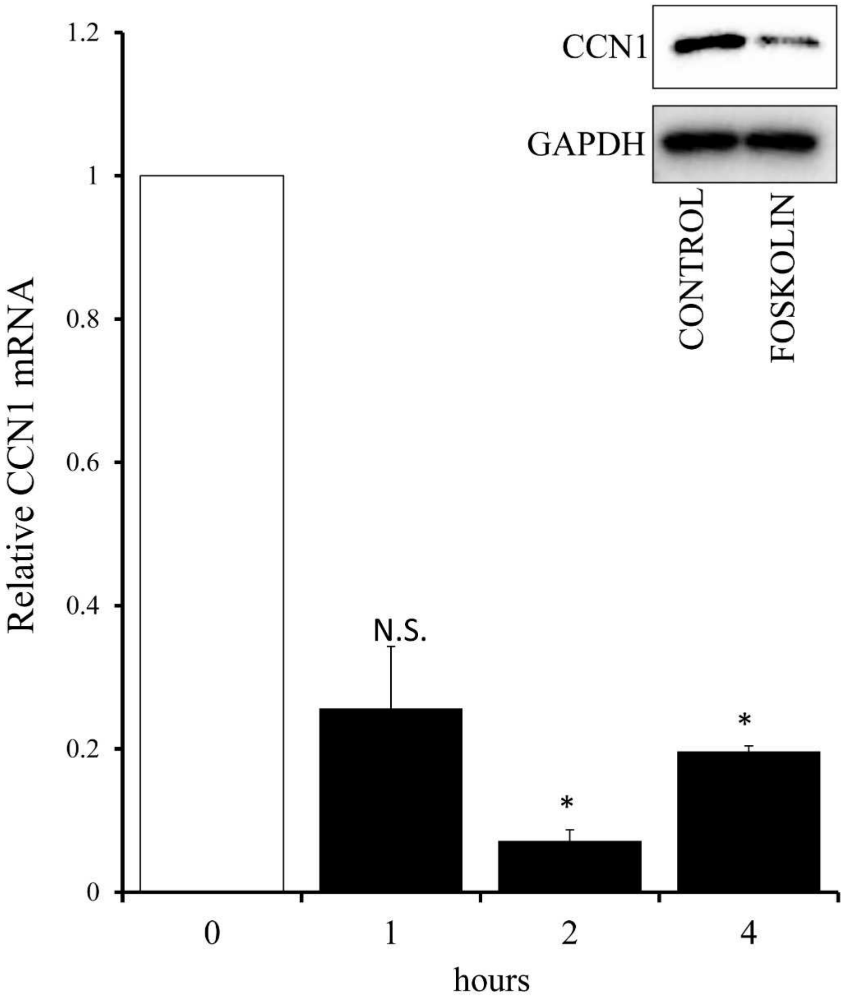

Supplement Figure S1: Effect of forskolin on CCN1 mRNA and protein levels in human aortic VSMC.

CCN1 mRNA levels in Human aortic VSMC stimulated with 10 µM forskolin for the indicated times. CCN1 protein was analysed by western blotting after 8 hours stimulation with 10 µM forskolin (inset).

Figure S2

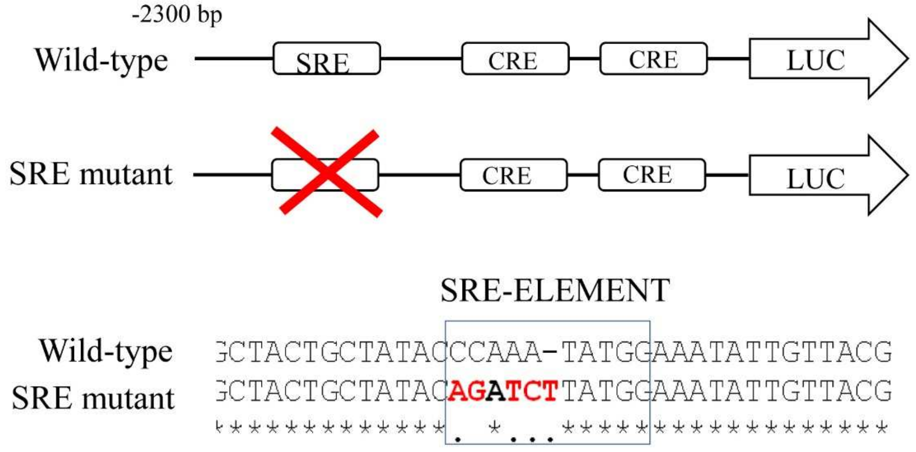

Supplement Figure S2

Schematic representation of the wild-type and SRE mutated CCN1 promoter luciferase reporter vector.

Figure S3

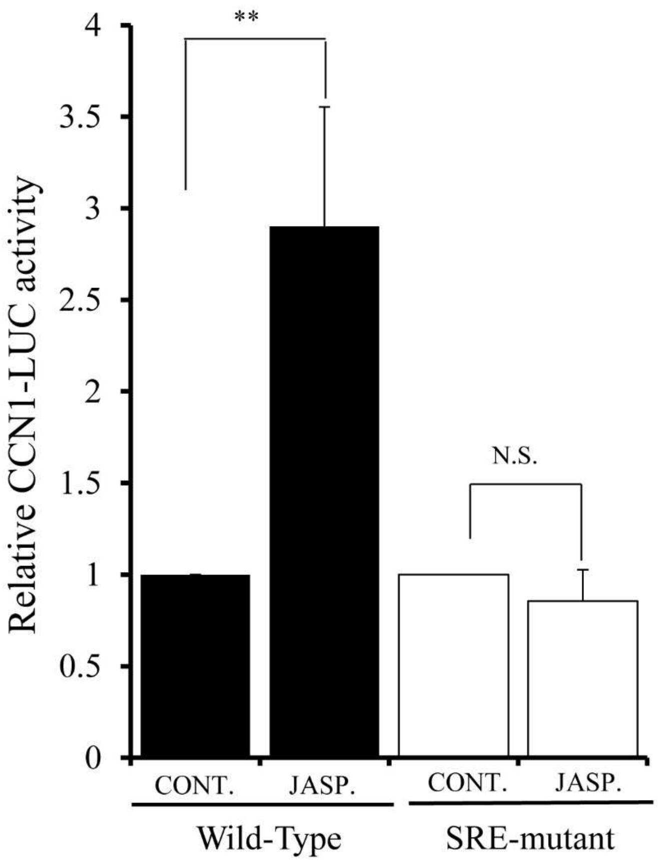

Supplement Figure S3

Activity of wild type and SRE-mutated CCN1 promoter after a 2 hour stimulation with 0.5 µM jasplakinolide**.**

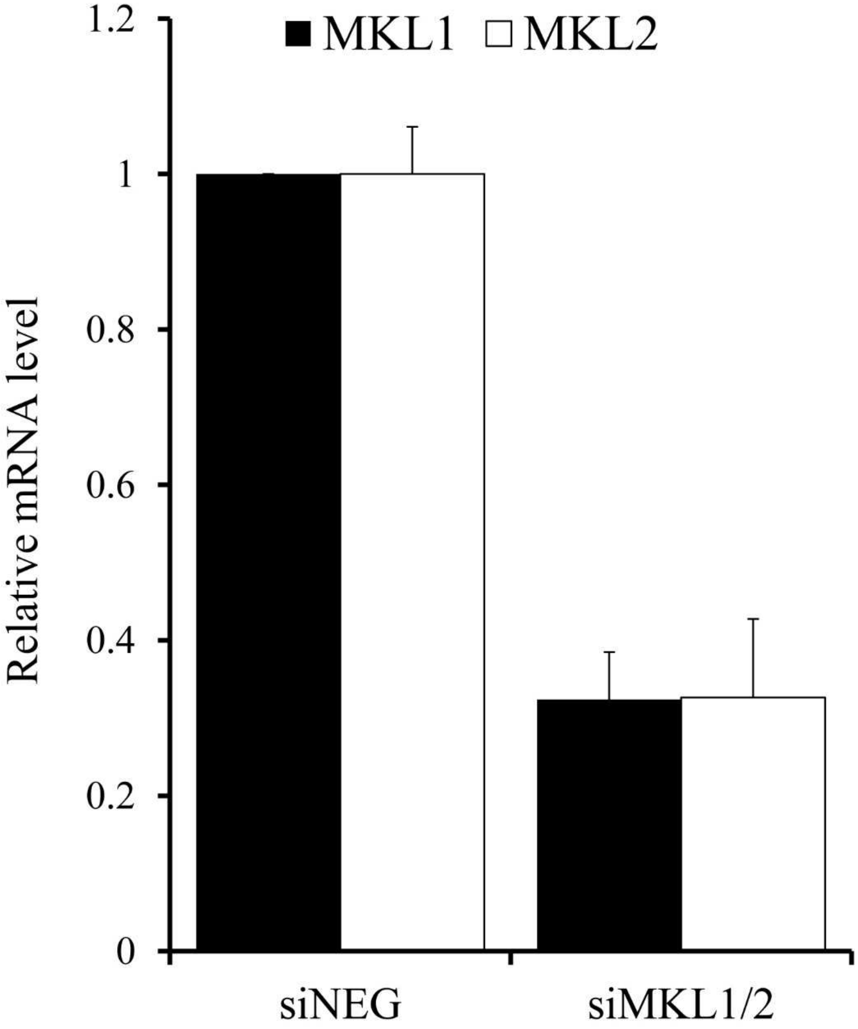

Supplement Figure S4

MKL1 and MKL2 mRNA levels in cells transfected with siRNA targeting MKL1 and MKL2.
